# Approach to Charcot Neuroarthropathy of the Great Toe: A Case Report

**DOI:** 10.7759/cureus.71368

**Published:** 2024-10-13

**Authors:** Pradeep Moonot, Prashant Pawar

**Affiliations:** 1 Orthopaedics, Mumbai Knee Foot Ankle Clinic, Mumbai, IND; 2 Orthopaedics, Dr. D.Y.Patil Medical College, Hospital and Research Centre, Dr. D.Y.Patil Vidyapeeth (Deemed to be University), Pune, IND

**Keywords:** charcot neuroarthropathy, consolidation, great toe, interphalangeal joint, k-wire fixation

## Abstract

Charcot neuroarthropathy (CN) is a chronic progressive debilitating disease affecting joints, bone and soft tissue of an insensate limb, usually seen in patients with diabetes. CN of the great toe is rare or it may be associated with CN of other joints. Only a few cases have been reported on the CN of the great toe. Stabilization and offloading is the primary aim of the treatment of CN. The present case report highlights the presentation, diagnosis and management of CN of the great toe. A 56-year-old male patient with diabetes presented to our outpatient department with post-traumatic swelling of the great toe with blackish discoloration and scanty, purulent discharging sinus. Based on the clinical and radiological findings, it was diagnosed to be the CN of the great toe, which was stabilized with a Kirschner wire. Clinical improvement and new bone formation were seen and the great toe was stabilized in acceptable alignment. Diagnosing CN of the great toe is challenging and needs both clinical and radiological evaluation. Stabilization with a single Kirschner wire is a simple, low-cost procedure, which can be done under a digital block in a minor operation theatre. Immediate mobilization and weight-bearing are allowed with good radiological and functional outcomes.

## Introduction

Charcot neuroarthropathy (CN) is a chronic, progressive, debilitating disease affecting joints, bone and soft tissue of the insensate limb, usually seen in patients with diabetes mellitus. The prevalence of CN in diabetic patients varies between 0.4% to 13% [1]. The prevalence of diabetes mellitus in India is higher than in the West and is gaining the status of a potential epidemic [2,3]. According to a recent survey by the International Diabetes Federation, diabetes prevalence is estimated to be 9.6% in 2021 and is projected to increase to 10.4% by 2030 [4]. Thus, the number of patients with CN is expected to rise in the coming decade. The most common location for CN is the midfoot (59%), followed by the ankle (29%), and then other parts of the skeleton [5]. Isolated CN of the great toe is rare or may be associated with CN of other joints [6,7]. To date, few cases have been reported regarding isolated CN of the great toe. In 1998, Beals and Manoli treated two patients with isolated CN of the great toe with stiff and hard-soled shoes for four months [6]. Hard and stiff soles can cause non-healing ulcers in CN patients with insensate feet. Apart from Beals and Manoli, the literature on the management of the isolated CN of the great toe is scarce. Over the past few decades, there have been advances in the management principles and techniques for CN [8]. The treatment of the CN aims to offload and stabilize the affected joint to avoid deformity and ulcer formation. The present case report highlights the presentation, the diagnostic challenges and the use of simple technique for managing isolated CN of the great toe. 

## Case presentation

**Figure 1:**
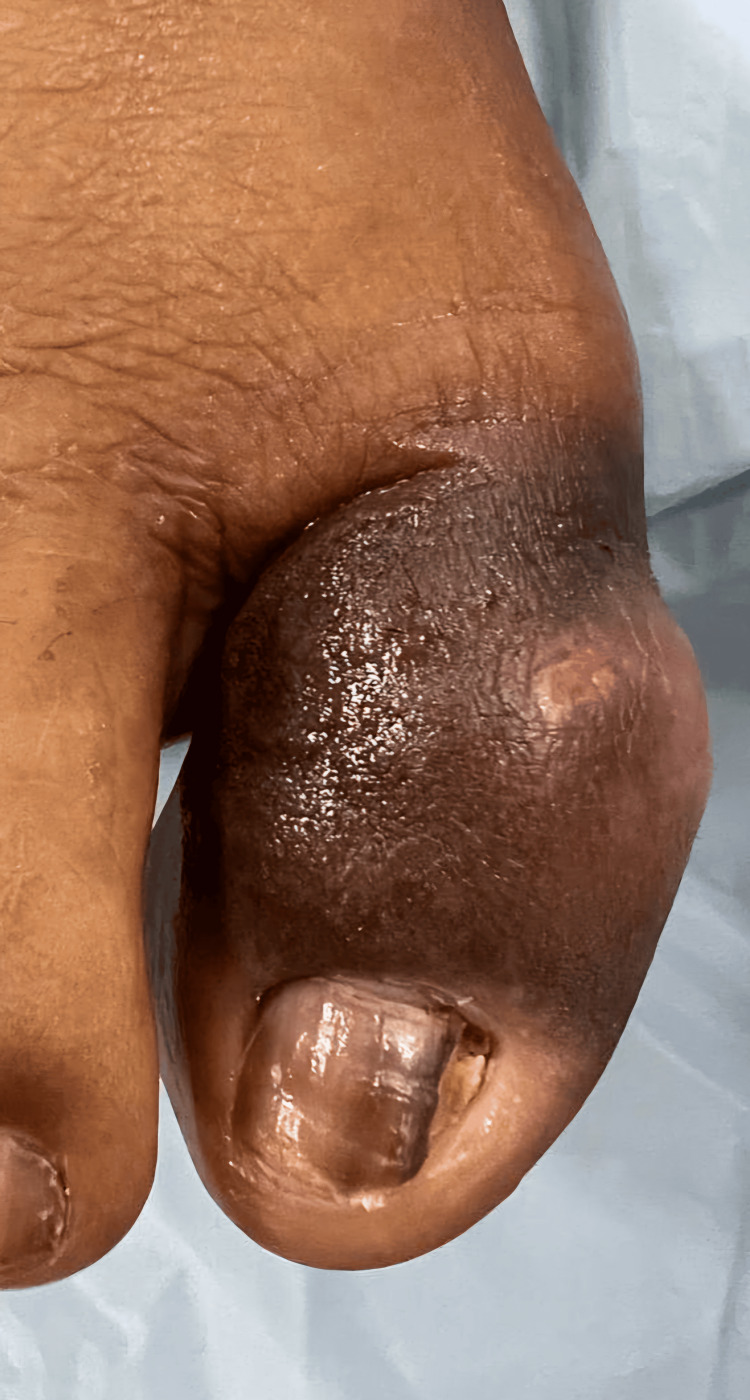
The right great toe appears to be swollen, erythematous and with a blackish discolouration.

**Figure 2:**
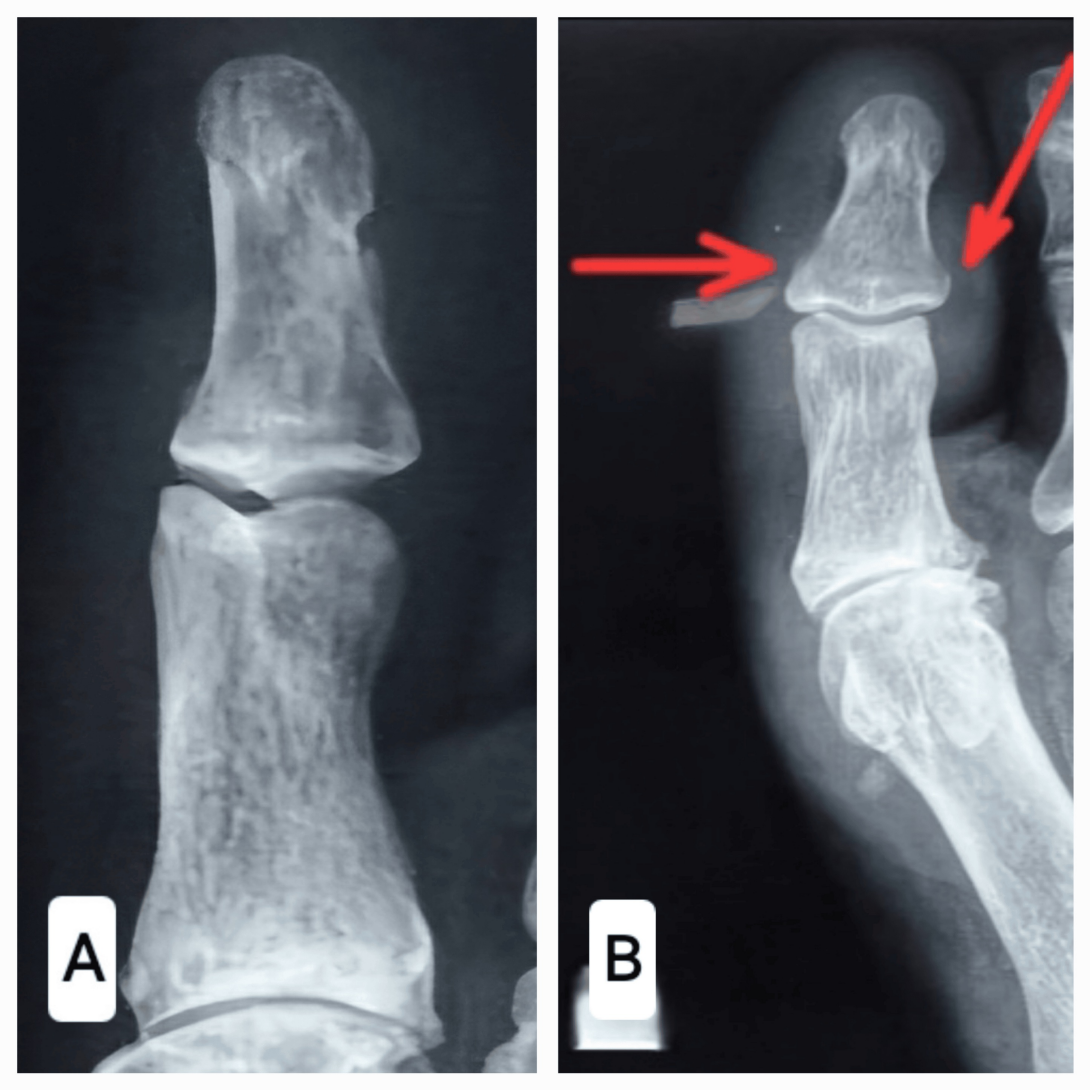
The plain radiograph of the affected great toe. A: The proximal and distal phalanx in the great toe appears to be normal. B: The distal phalanx of the affected toe shows post-traumatic cortical breach (red arrow).

A 56-year-old male presented to our outpatient department with a four-week history of swelling, blackish discolouration, discharging sinus and warmth over the great toe in the right foot (Figure 1). He was a known case of Type 2 diabetes mellitus for the last five years and was on tablet Metformin 500mg twice a day for diabetes. He had a trivial trauma to his great toe four weeks prior, and the radiograph revealed a cortical breach of the distal phalanx (Figure 2B, red arrows). The referring physician was primarily treating him for post-traumatic osteomyelitis of the great toe and advised him to debridement/amputation of the great toe, for which he sought a second opinion from us.

Physical examination revealed a swollen, erythematous, warm right great toe with scanty, purulent discharging sinus on the dorsal aspect (Figure 1). The prominent swelling was painless and non-tender on palpation. In addition, the swelling was gravity-dependent, which diminished with elevation of the foot. The range of motion of the interphalangeal (IP) joint was associated with crepitus, while the motion at the metatarsophalangeal joint was normal. The girth of the great toe at the IP joint was 12cm, as that of 9cm on the normal side. The delicate, crude touch, temperature, joint position, and vibration sensation were diminished in both feet below the ankle level. The flexion and extension at the IP joint were weak compared to the contralateral limb. Deformity or non-healing ulcer was absent. Peripheral pulses were palpable and symmetric on both sides. No history or signs were present suggestive of leprosy, poliomyelitis, spinal cord lesions and inflammatory arthritis. The laboratory investigation results are given in Table 1.

**Table 1:** Laboratory investigations HbA1c: glycosylated haemoglobin

Laboratory test	Observed Value	Normal range
Haemoglobin	13.5 g/dl	14-18 g/dl
Total leucocyte count	6500 cells/mm^3^	4000–11,000 cells/mm^3^
Creatinine	1.06 mg/dl	0.7-1.3 mg/dl
HbA1c	6.2%	<5.7%
Erythrocyte sedimentation rate	37 mm/h	<20 mm/h
C-reactive protein	0.9 mg/dl	0.9 mg/dl

**Figure 3:**
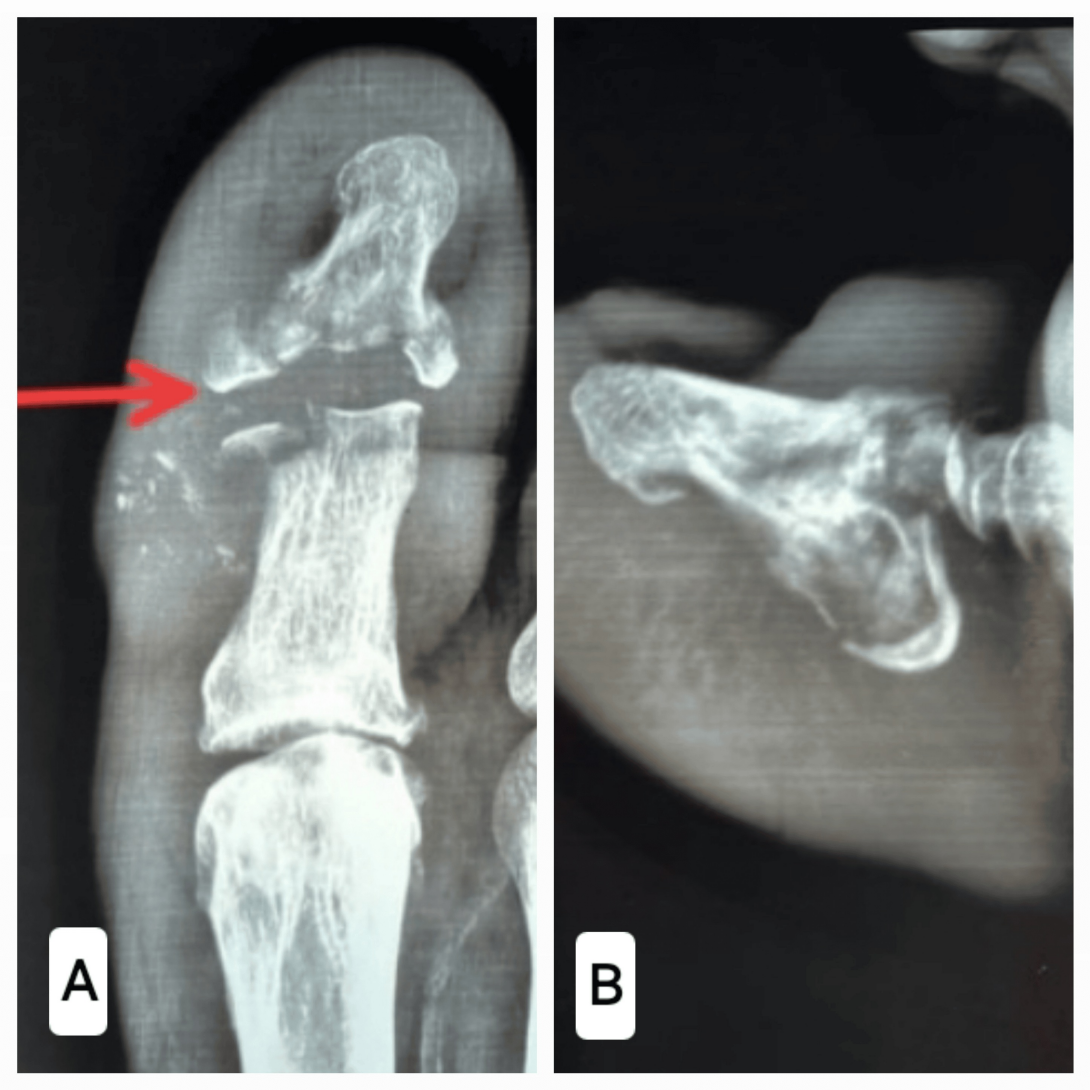
Plain radiograph of the affected great toe. A: The antero-posterior view showing intra-articular fracture of distal phalanx with fragmentation and bony debris. B: The lateral view showing intra-articular fracture of distal phalanx.

**Figure 4:**
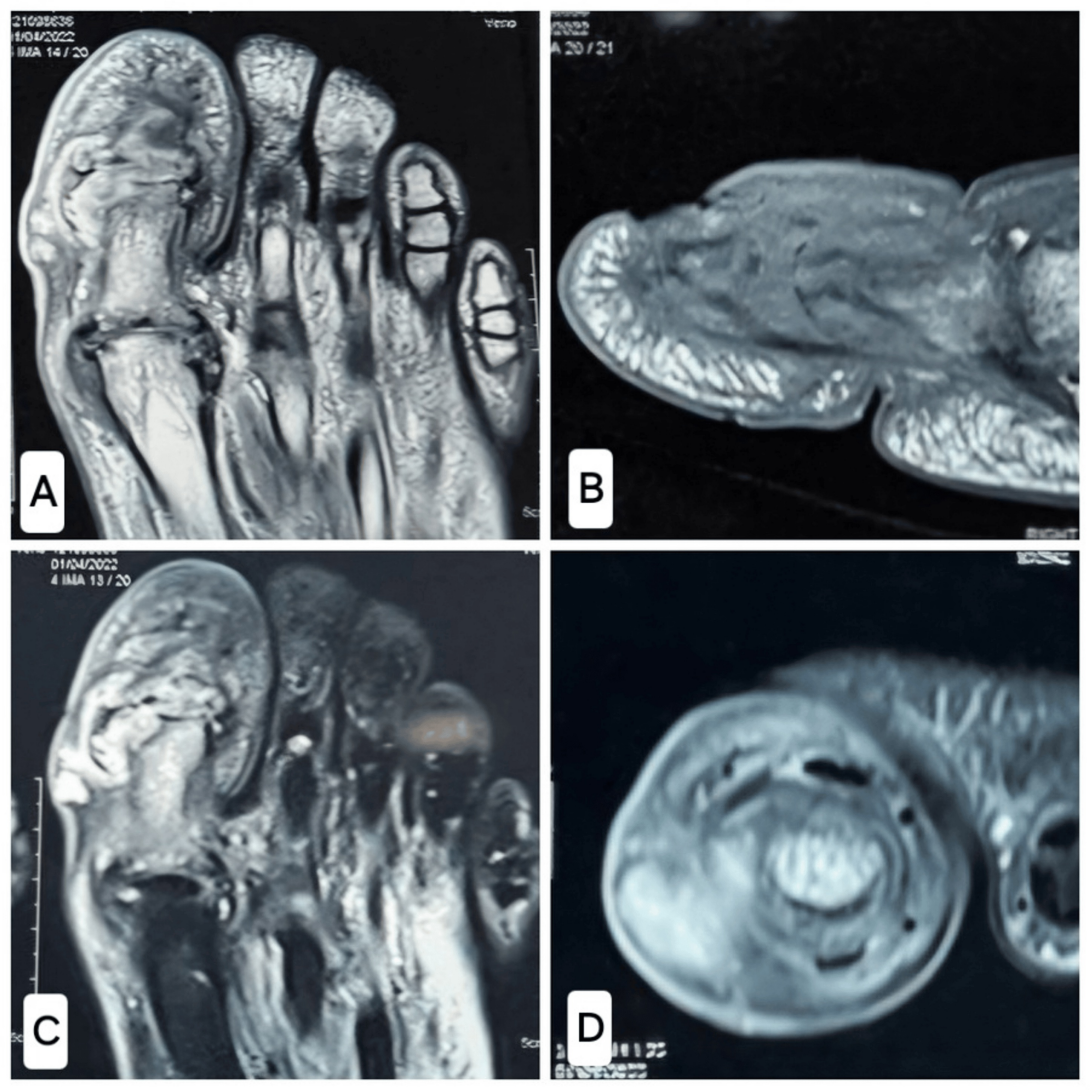
MRI of the affected great toe. A, B: T1 images of the affected great toe. C, D: T2 images of the affected great toe.

Radiographs revealed a comminutive intra-articular fracture of the distal phalanx with the fragmentation of the interphalangeal (IP) joint and bony debris in the IP joint (Figure 3). The magnetic resonance imaging of the great toe (Figure 4) revealed marrow oedema in the proximal and distal phalanges, extensively fragmented fracture in the proximal half of the distal phalanx of the great toe, a minor fracture in the distal articular aspect of the proximal phalanx, widening of the IP joint, a small localized pocket of fluid on the medial part of the IP joint of the great toe, disruption of the collateral ligaments of the IP common and patchy oedema in various plantar muscles underneath the metatarsals. Based on the clinical condition and imaging, the diagnosis of CN of the IP joint was confirmed (Eichenholtz Stage I, Sanders and Frykberg Type I).

**Figure 5:**
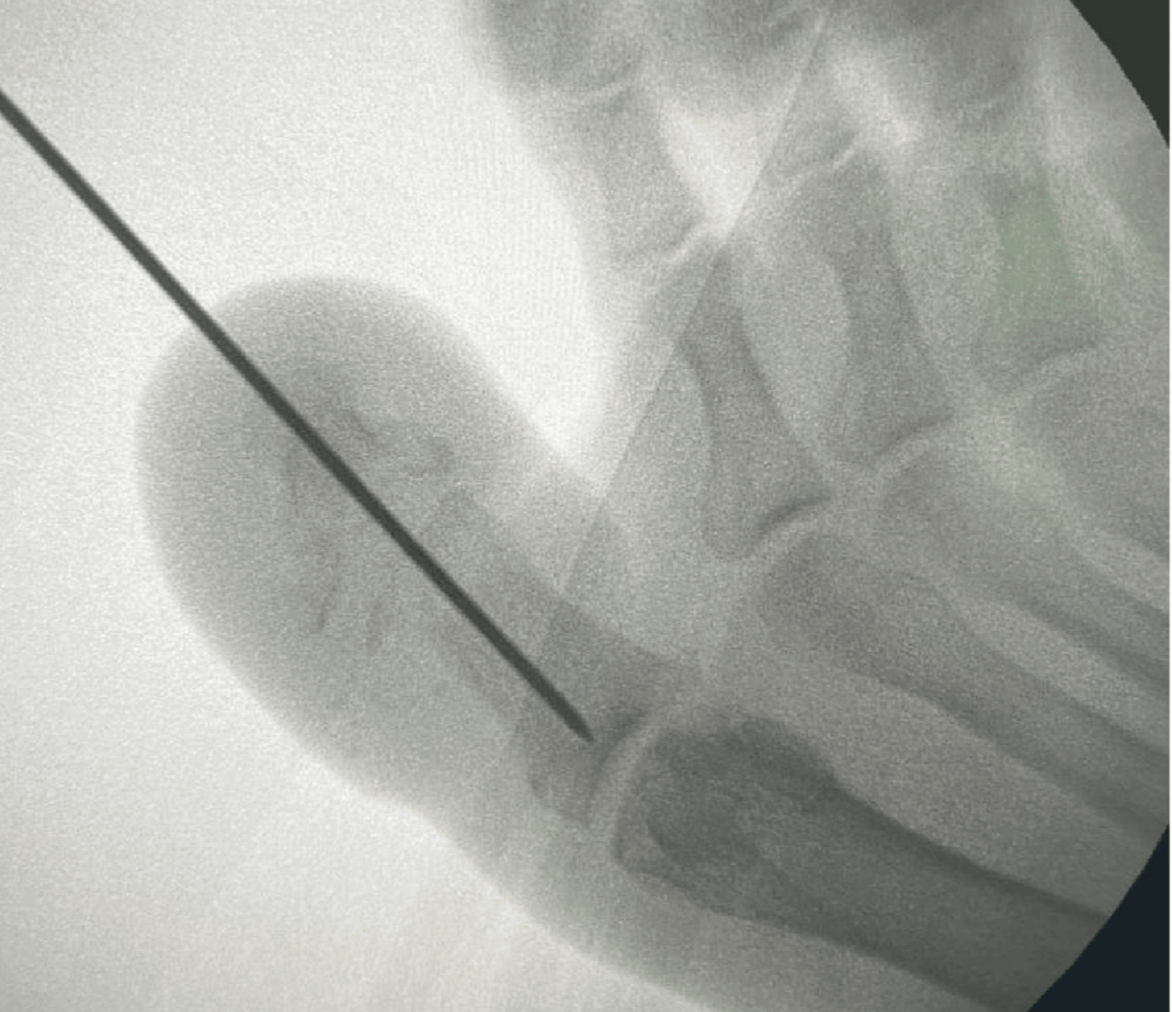
The Kirschner wire insertion in the proximal and distal phalanx under image intensifier.

We informed the patient about the diagnosis and the need to stabilize the IP joint to avoid deformity and ulceration. With due consent, the procedure was planned in the minor operating room under the digital nerve block using 2% Lidocaine HCl. Scrubbing and draping of the operative foot were done. The IP joint was transfixed with a single 1.8mm Kirschner wire. The wire was passed from the tip of the distal phalanx, transfixing the IP joint into the proximal phalanx under the image intensifier's guidance (Figure 5). The dressing was done, and the patient was discharged with a prescription of tablet paracetamol 650mg thrice a day. Postoperatively, the patient was mobilized with forefoot offloading footwear on the same day.

**Figure 6:**
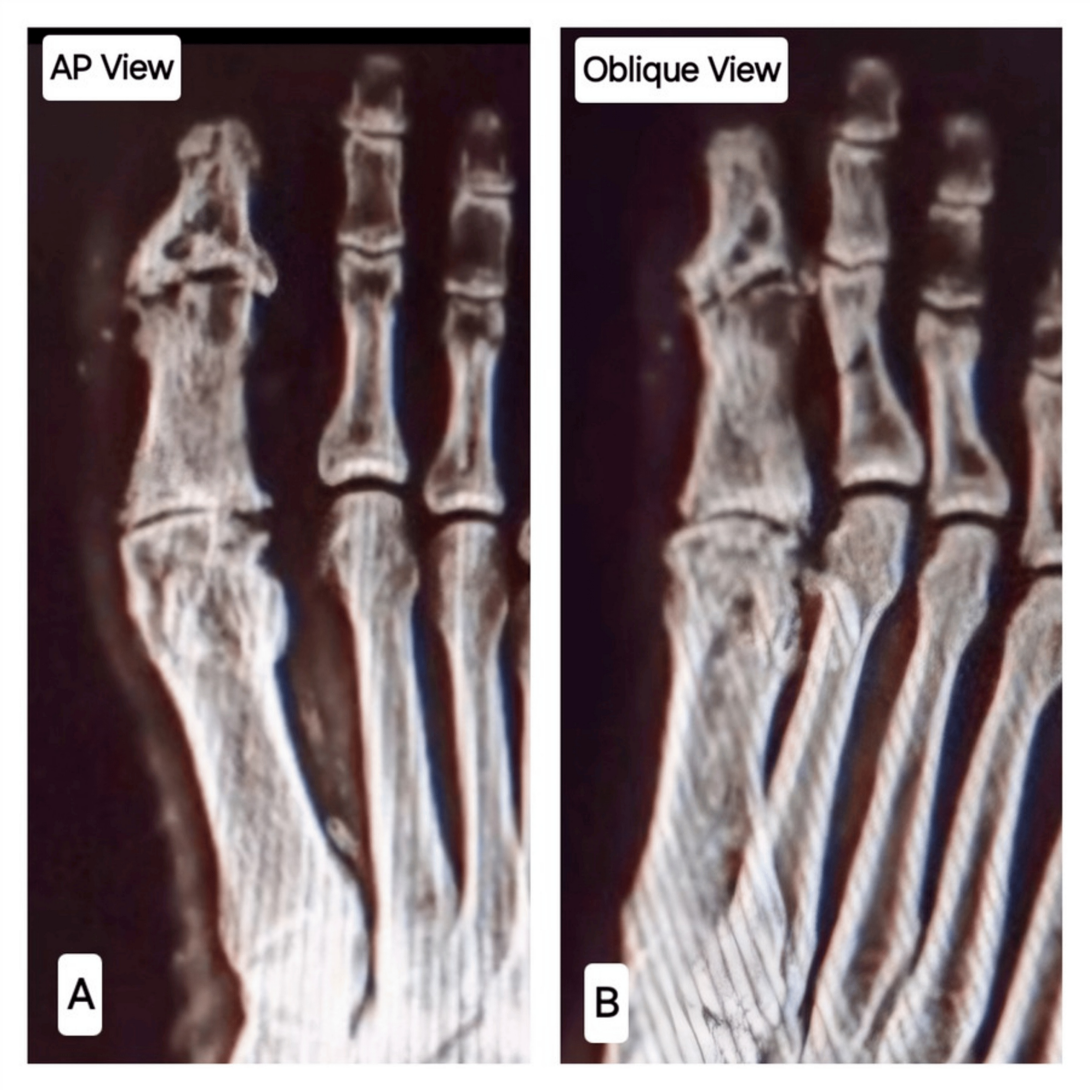
Plain radiograph of the affected toe (post-operative at six months follow-up)

**Figure 7:**
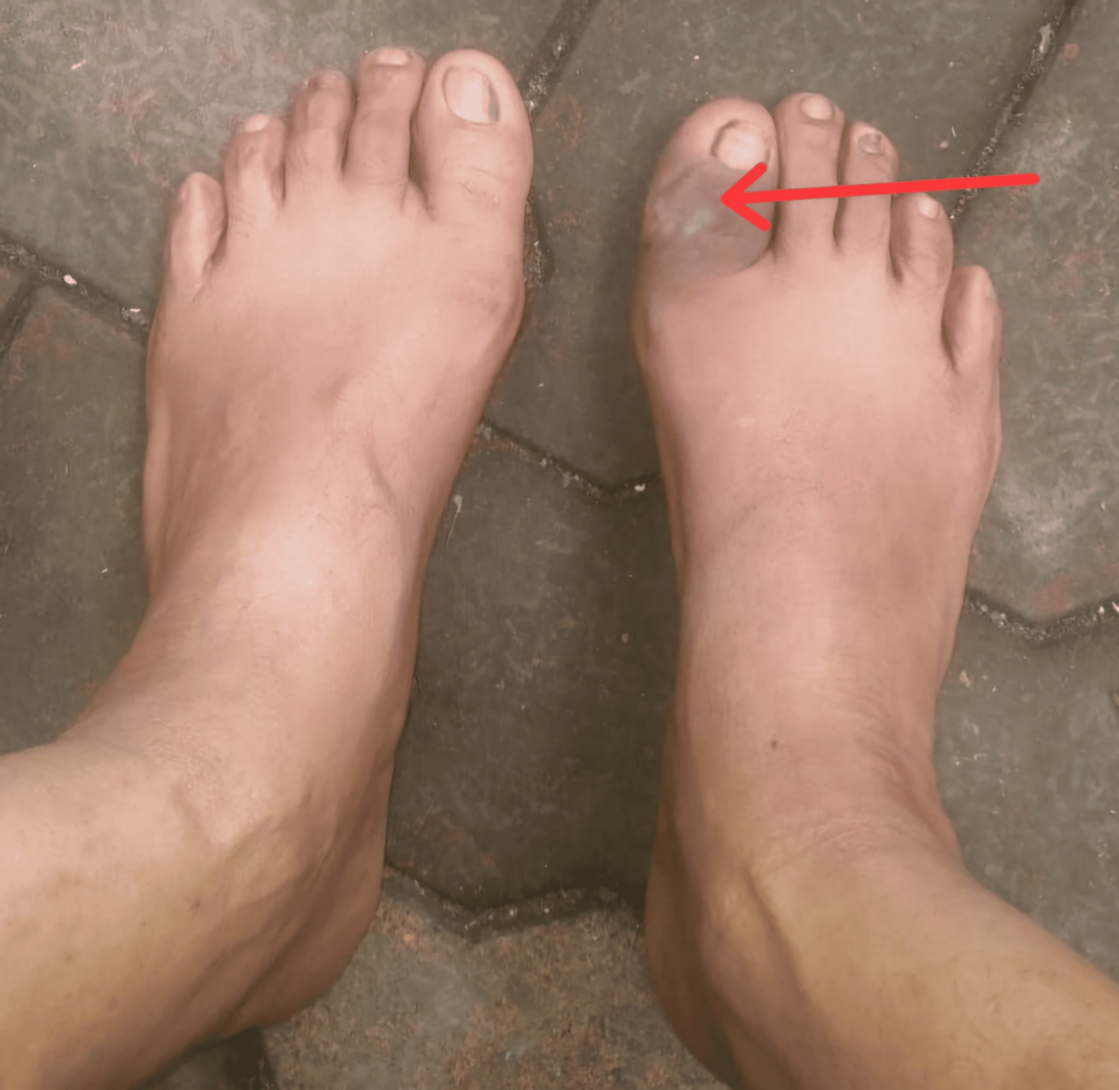
Clinical image of the affected great toe showing normal alignment at six months follow-up.

The bandage was done every fifth day in the outpatient department, and the swelling was evaluated. The swelling, warmth and erythema subsequently diminished over eight weeks, and girth of the great toe reduced to the size of the opposite toe (9cm). The Kirschner wire was removed in the outpatient department (OPD) at six weeks. Serial radiographs taken during follow-up, revealed absorption of the bony debris, new bone formation and fusion of large bony fragments, along with smoothening and rounding of the edges. In Stage III, consolidation of the distal phalanx was seen as per the Eichenholtz classification (Figure 6). Clinically, the great toe was stabilized at the end of six months with no deformity or ulcer formation (Figure 7). The patient is weight-bearing comfortably without new ulcer formation after two years post-surgery.

## Discussion

The appropriate diagnosis of the CN of the great toe, which is rare, is challenging and is based on clinical and radiological parameters. The acute phase of CN mimics infection, which needs accurate early diagnosis and appropriate treatment. A delay in the diagnosis and treatment may result in deformity, non-healing ulcer and amputation. In our case, differential diagnoses of cellulitis, osteomyelitis and inflammatory arthropathy were considered. The CN classically presents as painless and non-tender swelling with erythema and warmth over a localized region in a neuropathic limb, which is against the findings of infection or inflammatory swelling. Elevation of the involved lower limb and monitoring the resolution of the swelling is an important test to differentiate between CN and infection/cellulitis. The decrease in swelling is suggestive of CN, while persistent swelling and erythema support infection/cellulitis [9,10]. Also, infection is rare without penetrating trauma.

It is often challenging to differentiate CN from osteomyelitis radiologically. Radiological findings seen are bone marrow and soft tissue oedema, fluid collection, subchondral sclerosis, narrowing of the joint space, bony resorption and fractures with subluxation. The main findings, which help to differentiate CN on MRI, are periarticular oedema in CN as opposed to the diffuse bony involvement in osteomyelitis. The subchondral cyst is usually seen in the CN, which disappears in the infection/ osteomyelitis, and the presence of the intra-articular bodies rules out osteomyelitis; the intra-articular bodies are disintegrated in osteomyelitis due to surrounding inflammation [11].

In CN, autonomic neuropathy causes vasoregulatory failure, leading to increased blood flow to the bone and osteoclast transport to the affected bone. The increased osteoclastic activity causes local bone resorption, osteoporosis, fractures in weight-bearing bones and joint dislocation [12]. Placing weight on a numb limb with weakened bones may cause misalignment, bone fragmentation and collapse, deformities and the development of neuropathic ulcers [13].

CN management aims to immobilize and offload the active region in the acute phase to avoid deformity and ulceration, which is suitable for ambulation. The immobilization and resting of the joint help in the reduction of both hyperaemia and bone resorption. Immobilization of the great toe can be done through various methods. By non-surgical methods, it can be done by splinting or buddy strapping. In 1998, Beals and Manoli conservatively treated two patients with CN of the great toe by recommending stiff and hard-soled shoes for four months [6]. However, strapping and splinting may not be adequate immobilization and may lead to deformity and ulcer formation. The repeated change of buddy strapping can be uncomfortable for the patient and cause repeated micro-movements during the change, leading to a more extended recovery period. Also, the patient needs to be non-weight-bearing for longer with conservative methods. Surgically, the great toe can be stabilized with a mini-external fixator or Kirschner wires. Stabilization with a mini-external fixator is cumbersome and inconvenient to the patient. It requires regular pin tract dressing and makes it challenging to wear footwear. We describe a simple technique to immobilize the IP joint with a single Kirschner wire under a digital nerve block, allowing weight-bearing with forefoot offloading shoes from the same day. As seen in our case, as soon as the IP joint was stabilized, the swelling was reduced and new bone formation was observed. The affected bone and joint consolidated in a neutral position, thus avoiding the possibility of IP joint deformity and ulcer formation.

## Conclusions

Early detection and stabilization of the Charcot neuropathy is essential to avoid the bony deformity and joint preservation. Differentiating Charcot neuropathy from acute osteomyelitis is challenging and needs both clinical and radiological consideration. Early offloading and stabilizing of the affected joint helps to maintain the alignment of the limb during the consolidation stage. Charcot neuropathy of the great toe is rare, and a high degree of suspicion is needed to diagnose it. It's essential to stabilize the great toe, which helps to maintain the alignment and prevents further complications and amputation. Stabilization with a single Kirschner wire is a simple, low-cost procedure, which can be done under a digital nerve block in the minor operation theatre, with good radiological and functional outcomes.
